# Supplementary material for: Examining the interplay between mental health indicators and quality of life measures among first-year law students: a cross-sectional study
Source: PeerJ. 2024 Nov 11;12:e18245. doi: 10.7717/peerj.18245 (PMC11562776; doi:10.7717/peerj.18245)
Supplement: Supplemental Information 3 — Correlation matrices and descriptive statistics for the variables investigated. [file peerj-12-18245-s003.docx]

| **Descriptive Statistics** | | | |
| --- | --- | --- | --- |
|  | Mean | Std. Deviation | N |
| DEPRESSION | 5.9733 | 4.21379 | 75 |
| STRESS | 7.8133 | 4.80341 | 75 |
| ANXIETY | 6.1733 | 4.57778 | 75 |
| FS | 3.5200 | 1.45528 | 75 |
| EIFI | 20.7333 | 10.59619 | 75 |
| MBPAQTotal | 8.4339 | 1.35188 | 75 |
| CDRSActual | 4.2133 | 1.90514 | 75 |
| WHOD3 | 12.4667 | 2.33269 | 75 |

| **Correlations** | | | | | | | | | |
| --- | --- | --- | --- | --- | --- | --- | --- | --- | --- |
|  | | DEPRESSION | STRESS | ANXIETY | FS | EIFI | MBPAQTotal | CDRSActual | WHOD3 |
| DEPRESSION | Pearson Correlation | 1 | .738^**^ | .805^**^ | .121 | .267^*^ | -.314^**^ | .186 | -.433^**^ |
|  | Sig. (2-tailed) |  | .000 | .000 | .300 | .021 | .006 | .110 | .000 |
|  | N | 75 | 75 | 75 | 75 | 75 | 75 | 75 | 75 |
| STRESS | Pearson Correlation | .738^**^ | 1 | .837^**^ | .095 | .465^**^ | -.324^**^ | .251^*^ | -.639^**^ |
|  | Sig. (2-tailed) | .000 |  | .000 | .416 | .000 | .005 | .030 | .000 |
|  | N | 75 | 75 | 75 | 75 | 75 | 75 | 75 | 75 |
| ANXIETY | Pearson Correlation | .805^**^ | .837^**^ | 1 | .067 | .548^**^ | -.290^*^ | .287^*^ | -.595^**^ |
|  | Sig. (2-tailed) | .000 | .000 |  | .565 | .000 | .012 | .013 | .000 |
|  | N | 75 | 75 | 75 | 75 | 75 | 75 | 75 | 75 |
| FS | Pearson Correlation | .121 | .095 | .067 | 1 | .099 | .181 | .008 | -.116 |
|  | Sig. (2-tailed) | .300 | .416 | .565 |  | .400 | .120 | .944 | .321 |
|  | N | 75 | 75 | 75 | 75 | 75 | 75 | 75 | 75 |
| EIFI | Pearson Correlation | .267^*^ | .465^**^ | .548^**^ | .099 | 1 | .026 | .400^**^ | -.308^**^ |
|  | Sig. (2-tailed) | .021 | .000 | .000 | .400 |  | .824 | .000 | .007 |
|  | N | 75 | 75 | 75 | 75 | 75 | 75 | 75 | 75 |
| MBPAQTotal | Pearson Correlation | -.314^**^ | -.324^**^ | -.290^*^ | .181 | .026 | 1 | -.040 | .314^**^ |
|  | Sig. (2-tailed) | .006 | .005 | .012 | .120 | .824 |  | .732 | .006 |
|  | N | 75 | 75 | 75 | 75 | 75 | 75 | 75 | 75 |
| CDRSActual | Pearson Correlation | .186 | .251^*^ | .287^*^ | .008 | .400^**^ | -.040 | 1 | -.135 |
|  | Sig. (2-tailed) | .110 | .030 | .013 | .944 | .000 | .732 |  | .247 |
|  | N | 75 | 75 | 75 | 75 | 75 | 75 | 75 | 75 |
| WHOD3 | Pearson Correlation | -.433^**^ | -.639^**^ | -.595^**^ | -.116 | -.308^**^ | .314^**^ | -.135 | 1 |
|  | Sig. (2-tailed) | .000 | .000 | .000 | .321 | .007 | .006 | .247 |  |
|  | N | 75 | 75 | 75 | 75 | 75 | 75 | 75 | 75 |
| **. Correlation is significant at the 0.01 level (2-tailed). | | | | | | | | | |
| *. Correlation is significant at the 0.05 level (2-tailed). | | | | | | | | | |

To provide anonymized raw data for review and publication as per your open data policy, here is the formatted data from the file "Appendix Correlations.docx". This includes descriptive statistics and correlations. The data was analyzed using SPSS, version 23.0. Reviewers can use SPSS or any other statistical software that supports similar data formats to verify the statistical analyses. This anonymized dataset can be opened and reviewed using SPSS version 23.0 or other compatible statistical software. The file includes correlation matrices and descriptive statistics for the variables investigated.
