# Supplementary material for: Examining the interplay between mental health indicators and quality of life measures among first-year law students: a cross-sectional study
Source: PeerJ. 2024 Nov 11;12:e18245. doi: 10.7717/peerj.18245 (PMC11562776; doi:10.7717/peerj.18245)
Supplement: Supplemental Information 4 — The data includes regression analysis results with unstandardized and standardized coefficients, t-values, and significance levels for multiple models. The software required to view and analyze the data is SPSS, version 23.0 or any compatible version. Regression Analysis Results Dependent Variable: Depression (DEPRESIA) Model 1: Independent Variable: Stress (STRES) Coefficients: Constant: B = 0.914, Std. Error = 0.634, t = 1.441, Sig. = 0.154 STRES: B = 0.648, Std. Error = 0.069, Beta = 0.738, t = 9.349, Sig. = 0.000 Model 2: Independent Variable: Anxiety (ANXIETATE) Coefficients: Constant: B = 1.398, Std. Error = 0.490, t = 2.854, Sig. = 0.006 ANXIETATE: B = 0.741, Std. Error = 0.064, Beta = 0.805, t = 11.600, Sig. = 0.000 Model 3: Independent Variable: Emotional Intelligence and Family Influence (EIFI) Coefficients: Constant: B = 3.771, Std. Error = 1.043, t = 3.616, Sig. = 0.001 EIFI: B = 0.106, Std. Error = 0.045, Beta = 0.267, t = 2.368, Sig. = 0.021 Model 4: Independent Variable: Physical Activity Total (MBPAQTotal) Coefficients: Constant: B = 14.235, Std. Error = 2.958, t = 4.813, Sig. = 0.000 MBPAQTotal: B = -0.980, Std. Error = 0.346, Beta = -0.314, t = -2.829, Sig. = 0.006 Model 5: Independent Variable: WHO Disability Assessment Schedule 3.0 (WHOD3) Coefficients: Constant: B = 15.728, Std. Error = 2.416, t = 6.509, Sig. = 0.000 WHOD3: B = -0.782, Std. Error = 0.191, Beta = -0.433, t = -4.106, Sig. = 0.000 Dependent Variable: Stress (STRES) Model 1: Independent Variable: Depression (DEPRESIE) Coefficients: Constant: B = 2.787, Std. Error = 0.656, t = 4.245, Sig. = 0.000 DEPRESIE: B = 0.841, Std. Error = 0.090, Beta = 0.738, t = 9.349, Sig. = 0.000 Model 2: Independent Variable: Anxiety (ANXIETATE) Coefficients: Constant: B = 2.390, Std. Error = 0.515, t = 4.642, Sig. = 0.000 ANXIETATE: B = 0.879, Std. Error = 0.067, Beta = 0.837, t = 13.084, Sig. = 0.000 Model 3: Independent Variable: Emotional Intelligence and Family Influence (EIFI) Coefficients: Constant [file peerj-12-18245-s004.docx]

Appendix CALCULATION OF REGRESSIONS

Here is the anonymized raw data from the " Appendix CALCULATION OF REGRESSIONS.docx" document for review and publication. The statistical analysis was conducted using SPSS, version 23.0.

Regression Analysis Results

Dependent Variable: Depression (DEPRESIA)

Model 1:

Independent Variable: Stress (STRES)

Coefficients:

Constant: B = 0.914, Std. Error = 0.634, t = 1.441, Sig. = 0.154

STRES: B = 0.648, Std. Error = 0.069, Beta = 0.738, t = 9.349, Sig. = 0.000

Model 2:

Independent Variable: Anxiety (ANXIETATE)

Coefficients:

Constant: B = 1.398, Std. Error = 0.490, t = 2.854, Sig. = 0.006

ANXIETATE: B = 0.741, Std. Error = 0.064, Beta = 0.805, t = 11.600, Sig. = 0.000

Model 3:

Independent Variable: Emotional Intelligence and Family Influence (EIFI)

Coefficients:

Constant: B = 3.771, Std. Error = 1.043, t = 3.616, Sig. = 0.001

EIFI: B = 0.106, Std. Error = 0.045, Beta = 0.267, t = 2.368, Sig. = 0.021

Model 4:

Independent Variable: Physical Activity Total (MBPAQTotal)

Coefficients:

Constant: B = 14.235, Std. Error = 2.958, t = 4.813, Sig. = 0.000

MBPAQTotal: B = -0.980, Std. Error = 0.346, Beta = -0.314, t = -2.829, Sig. = 0.006

Model 5:

Independent Variable: WHO Disability Assessment Schedule 3.0 (WHOD3)

Coefficients:

Constant: B = 15.728, Std. Error = 2.416, t = 6.509, Sig. = 0.000

WHOD3: B = -0.782, Std. Error = 0.191, Beta = -0.433, t = -4.106, Sig. = 0.000

Dependent Variable: Stress (STRES)

Model 1:

Independent Variable: Depression (DEPRESIE)

Coefficients:

Constant: B = 2.787, Std. Error = 0.656, t = 4.245, Sig. = 0.000

DEPRESIE: B = 0.841, Std. Error = 0.090, Beta = 0.738, t = 9.349, Sig. = 0.000

Model 2:

Independent Variable: Anxiety (ANXIETATE)

Coefficients:

Constant: B = 2.390, Std. Error = 0.515, t = 4.642, Sig. = 0.000

ANXIETATE: B = 0.879, Std. Error = 0.067, Beta = 0.837, t = 13.084, Sig. = 0.000

Model 3:

Independent Variable: Emotional Intelligence and Family Influence (EIFI)

Coefficients:

Constant: B = 3.446, Std. Error = 1.092, t = 3.154, Sig. = 0.002

EIFI: B = 0.211, Std. Error = 0.047, Beta = 0.465, t = 4.484, Sig. = 0.000

Model 4:

Independent Variable: Physical Activity Total (MBPAQTotal)

Coefficients:

Constant: B = 17.508, Std. Error = 3.361, t = 5.210, Sig. = 0.000

MBPAQTotal: B = -1.150, Std. Error = 0.393, Beta = -0.324, t = -2.921, Sig. = 0.005

Model 5:

Independent Variable: Actual Chronic Daily Stress (CDRSActual)

Coefficients:

Constant: B = 5.147, Std. Error = 1.319, t = 3.901, Sig. = 0.000

CDRSActual: B = 0.633, Std. Error = 0.286, Beta = 0.251, t = 2.216, Sig. = 0.030

Model 6:

Independent Variable: WHO Disability Assessment Schedule 3.0 (WHOD3)

Coefficients:

Constant: B = 24.206, Std. Error = 2.352, t = 10.292, Sig. = 0.000

WHOD3: B = -1.315, Std. Error = 0.185, Beta = -0.639, t = -7.089, Sig. = 0.000

Dependent Variable: Anxiety (ANXIETATE)

Model 1:

Independent Variable: Depression (DEPRESIE)

Coefficients:

Constant: B = 0.948, Std. Error = 0.550, t = 1.724, Sig. = 0.089

DEPRESIE: B = 0.875, Std. Error = 0.075, Beta = 0.805, t = 11.600, Sig. = 0.000

Model 2:

Independent Variable: Stress (STRES)

Coefficients:

Constant: B = -0.061, Std. Error = 0.558, t = -0.110, Sig. = 0.913

STRES: B = 0.798, Std. Error = 0.061, Beta = 0.837, t = 13.084, Sig. = 0.000

Model 3:

Independent Variable: Emotional Intelligence and Family Influence (EIFI)

Coefficients:

Constant: B = 1.261, Std. Error = 0.983, t = 1.283, Sig. = 0.204

EIFI: B = 0.237, Std. Error = 0.042, Beta = 0.548, t = 5.603, Sig. = 0.000

Model 4:

Independent Variable: Physical Activity Total (MBPAQTotal)

Coefficients:

Constant: B = 14.458, Std. Error = 3.239, t = 4.464, Sig. = 0.000

MBPAQTotal: B = -0.982, Std. Error = 0.379, Beta = -0.290, t = -2.590, Sig. = 0.012

Model 5:

Independent Variable: Actual Chronic Daily Stress (CDRSActual)

Coefficients:

Constant: B = 3.268, Std. Error = 1.244, t = 2.626, Sig. = 0.011

CDRSActual: B = 0.690, Std. Error = 0.269, Beta = 0.287, t = 2.560, Sig. = 0.013

Model 6:

Independent Variable: WHO Disability Assessment Schedule 3.0 (WHOD3)

Coefficients:

Constant: B = 20.727, Std. Error = 2.341, t = 8.853, Sig. = 0.000

WHOD3: B = -1.167, Std. Error = 0.185, Beta = -0.595, t = -6.323, Sig. = 0.000

Custom File Format Description

The data is stored in a Microsoft Word document format (.docx) and includes regression analysis results with unstandardized and standardized coefficients, t-values, and significance levels for multiple models. The software required to view and analyze the data is SPSS, version 23.0 or any compatible version.

**Variabila dependentă - DEPRESIA**

| **Coefficients^a^** | | | | | | |
| --- | --- | --- | --- | --- | --- | --- |
| Model | | Unstandardized Coefficients | | Standardized Coefficients | t | Sig. |
|  |  | B | Std. Error | Beta |  |  |
| 1 | (Constant) | .914 | .634 |  | 1.441 | .154 |
|  | STRES | .648 | .069 | .738 | 9.349 | .000 |
| a. Dependent Variable: DEPRESIE | | | | | | |

| **Coefficients^a^** | | | | | | |
| --- | --- | --- | --- | --- | --- | --- |
| Model | | Unstandardized Coefficients | | Standardized Coefficients | t | Sig. |
|  |  | B | Std. Error | Beta |  |  |
| 1 | (Constant) | 1.398 | .490 |  | 2.854 | .006 |
|  | ANXIETATE | .741 | .064 | .805 | 11.600 | .000 |
| a. Dependent Variable: DEPRESIE | | | | | | |

| **Coefficients^a^** | | | | | | |
| --- | --- | --- | --- | --- | --- | --- |
| Model | | Unstandardized Coefficients | | Standardized Coefficients | t | Sig. |
|  |  | B | Std. Error | Beta |  |  |
| 1 | (Constant) | 3.771 | 1.043 |  | 3.616 | .001 |
|  | EIFI | .106 | .045 | .267 | 2.368 | .021 |
| a. Dependent Variable: DEPRESIE | | | | | | |

| **Coefficients^a^** | | | | | | |
| --- | --- | --- | --- | --- | --- | --- |
| Model | | Unstandardized Coefficients | | Standardized Coefficients | t | Sig. |
|  |  | B | Std. Error | Beta |  |  |
| 1 | (Constant) | 14.235 | 2.958 |  | 4.813 | .000 |
|  | MBPAQTotal | -.980 | .346 | -.314 | -2.829 | .006 |
| a. Dependent Variable: DEPRESIE | | | | | | |

| **Coefficients^a^** | | | | | | |
| --- | --- | --- | --- | --- | --- | --- |
| Model | | Unstandardized Coefficients | | Standardized Coefficients | t | Sig. |
|  |  | B | Std. Error | Beta |  |  |
| 1 | (Constant) | 15.728 | 2.416 |  | 6.509 | .000 |
|  | WHOD3 | -.782 | .191 | -.433 | -4.106 | .000 |
| a. Dependent Variable: DEPRESIE | | | | | | |

**Variabila dependentă STRES**

| **Coefficients^a^** | | | | | | |
| --- | --- | --- | --- | --- | --- | --- |
| Model | | Unstandardized Coefficients | | Standardized Coefficients | t | Sig. |
|  |  | B | Std. Error | Beta |  |  |
| 1 | (Constant) | 2.787 | .656 |  | 4.245 | .000 |
|  | DEPRESIE | .841 | .090 | .738 | 9.349 | .000 |
| a. Dependent Variable: STRES | | | | | | |

| **Coefficients^a^** | | | | | | |
| --- | --- | --- | --- | --- | --- | --- |
| Model | | Unstandardized Coefficients | | Standardized Coefficients | t | Sig. |
|  |  | B | Std. Error | Beta |  |  |
| 1 | (Constant) | 2.390 | .515 |  | 4.642 | .000 |
|  | ANXIETATE | .879 | .067 | .837 | 13.084 | .000 |
| a. Dependent Variable: STRES | | | | | | |

| **Coefficients^a^** | | | | | | |
| --- | --- | --- | --- | --- | --- | --- |
| Model | | Unstandardized Coefficients | | Standardized Coefficients | t | Sig. |
|  |  | B | Std. Error | Beta |  |  |
| 1 | (Constant) | 3.446 | 1.092 |  | 3.154 | .002 |
|  | EIFI | .211 | .047 | .465 | 4.484 | .000 |
| a. Dependent Variable: STRES | | | | | | |

| **Coefficients^a^** | | | | | | |
| --- | --- | --- | --- | --- | --- | --- |
| Model | | Unstandardized Coefficients | | Standardized Coefficients | t | Sig. |
|  |  | B | Std. Error | Beta |  |  |
| 1 | (Constant) | 17.508 | 3.361 |  | 5.210 | .000 |
|  | MBPAQTotal | -1.150 | .393 | -.324 | -2.921 | .005 |
| a. Dependent Variable: STRES | | | | | | |

| **Coefficients^a^** | | | | | | |
| --- | --- | --- | --- | --- | --- | --- |
| Model | | Unstandardized Coefficients | | Standardized Coefficients | t | Sig. |
|  |  | B | Std. Error | Beta |  |  |
| 1 | (Constant) | 5.147 | 1.319 |  | 3.901 | .000 |
|  | CDRSActual | .633 | .286 | .251 | 2.216 | .030 |
| a. Dependent Variable: STRES | | | | | | |

| **Coefficients^a^** | | | | | | |
| --- | --- | --- | --- | --- | --- | --- |
| Model | | Unstandardized Coefficients | | Standardized Coefficients | t | Sig. |
|  |  | B | Std. Error | Beta |  |  |
| 1 | (Constant) | 24.206 | 2.352 |  | 10.292 | .000 |
|  | WHOD3 | -1.315 | .185 | -.639 | -7.089 | .000 |
| a. Dependent Variable: STRES | | | | | | |

**Variabila dependentă ANXIETATE**

| **Coefficients^a^** | | | | | | |
| --- | --- | --- | --- | --- | --- | --- |
| Model | | Unstandardized Coefficients | | Standardized Coefficients | t | Sig. |
|  |  | B | Std. Error | Beta |  |  |
| 1 | (Constant) | .948 | .550 |  | 1.724 | .089 |
|  | DEPRESIE | .875 | .075 | .805 | 11.600 | .000 |
| a. Dependent Variable: ANXIETATE | | | | | | |

| **Coefficients^a^** | | | | | | |
| --- | --- | --- | --- | --- | --- | --- |
| Model | | Unstandardized Coefficients | | Standardized Coefficients | t | Sig. |
|  |  | B | Std. Error | Beta |  |  |
| 1 | (Constant) | -.061 | .558 |  | -.110 | .913 |
|  | STRES | .798 | .061 | .837 | 13.084 | .000 |
| a. Dependent Variable: ANXIETATE | | | | | | |

| **Coefficients^a^** | | | | | | |
| --- | --- | --- | --- | --- | --- | --- |
| Model | | Unstandardized Coefficients | | Standardized Coefficients | t | Sig. |
|  |  | B | Std. Error | Beta |  |  |
| 1 | (Constant) | 1.261 | .983 |  | 1.283 | .204 |
|  | EIFI | .237 | .042 | .548 | 5.603 | .000 |
| a. Dependent Variable: ANXIETATE | | | | | | |

| **Coefficients^a^** | | | | | | |
| --- | --- | --- | --- | --- | --- | --- |
| Model | | Unstandardized Coefficients | | Standardized Coefficients | t | Sig. |
|  |  | B | Std. Error | Beta |  |  |
| 1 | (Constant) | 14.458 | 3.239 |  | 4.464 | .000 |
|  | MBPAQTotal | -.982 | .379 | -.290 | -2.590 | .012 |
| a. Dependent Variable: ANXIETATE | | | | | | |

| **Coefficients^a^** | | | | | | |
| --- | --- | --- | --- | --- | --- | --- |
| Model | | Unstandardized Coefficients | | Standardized Coefficients | t | Sig. |
|  |  | B | Std. Error | Beta |  |  |
| 1 | (Constant) | 3.268 | 1.244 |  | 2.626 | .011 |
|  | CDRSActual | .690 | .269 | .287 | 2.560 | .013 |
| a. Dependent Variable: ANXIETATE | | | | | | |

| **Coefficients^a^** | | | | | | |
| --- | --- | --- | --- | --- | --- | --- |
| Model | | Unstandardized Coefficients | | Standardized Coefficients | t | Sig. |
|  |  | B | Std. Error | Beta |  |  |
| 1 | (Constant) | 20.727 | 2.341 |  | 8.853 | .000 |
|  | WHOD3 | -1.167 | .185 | -.595 | -6.323 | .000 |
| a. Dependent Variable: ANXIETATE | | | | | | |
