# Supplementary material for: Examining the interplay between mental health indicators and quality of life measures among first-year law students: a cross-sectional study
Source: PeerJ. 2024 Nov 11;12:e18245. doi: 10.7717/peerj.18245 (PMC11562776; doi:10.7717/peerj.18245)
Supplement: Supplemental Information 5 [file peerj-12-18245-s005.docx]

STROBE Checklist for Observational Studies

# Title: Examining the interplay between mental health indicators and quality of life measures among first-year law students: a quantitative study

| Section | Item | Description | Reported on Page Number |
| --- | --- | --- | --- |
| Title and Abstract | 1. Title | a) Indicate the study’s design with a commonly used term in the title or the abstract | Page 1 |
| Title and Abstract | 2. Abstract | b) Provide in the abstract an informative and balanced summary of what was done and what was found | Page 1 |
| Introduction | 3. Background/rationale | Explain the scientific background and rationale for the investigation being reported | Page 2 |
| Introduction | 4. Objectives | State specific objectives, including any prespecified hypotheses | Page 3 |
| Methods | 5. Study Design | Present key elements of study design early in the paper | Page 4 |
| Methods | 6. Setting | Describe the setting, locations, and relevant dates, including periods of recruitment, exposure, follow-up, and data collection | Page 4 |
| Methods | 7. Participants | a) Cohort study—Give the eligibility criteria, and the sources and methods of selection of participants. Describe methods of follow-up | Page 5 |
| Methods |  | b) Case-control study—Give the eligibility criteria, and the sources and methods of case ascertainment and control selection. Give the rationale for the choice of cases and controls | Page 5 |
| Methods |  | c) Cross-sectional study—Give the eligibility criteria, and the sources and methods of selection of participants | Page 5 |
| Methods | 8. Variables | Clearly define all outcomes, exposures, predictors, potential confounders, and effect modifiers. Give diagnostic criteria, if applicable | Page 6 |
| Methods | 9. Data Sources/Measurement | For each variable of interest, give sources of data and details of methods of assessment (measurement). Describe comparability of assessment methods if there is more than one group | Page 6 |
| Methods | 10. Bias | Describe any efforts to address potential sources of bias | Page 6 |
| Methods | 11. Study Size | Explain how the study size was arrived at | Page 4 |
| Methods | 12. Quantitative Variables | Explain how quantitative variables were handled in the analyses. If applicable, describe which groupings were chosen and why | Page 6 |
| Methods | 13. Statistical Methods | a) Describe all statistical methods, including those used to control for confounding | Page 6 |
|  |  | b) Describe any methods used to examine subgroups and interactions | Page 6 |
|  |  |  |  |
|  |  | c) Describe any sensitivity analyses | Page 6 |
| Results | 14. Main Results | a) Give unadjusted estimates and, if applicable, confounder-adjusted estimates and their precision (e.g., 95% confidence interval). Make clear which confounders were adjusted for and why they were included | Page 6 |
|  |  | b) Report category boundaries when continuous variables were categorized | Page 7 |
| Discussion | 15. Key Results | Summarize key results with reference to study objectives | Page 8 |
| Discussion | 16. Interpretation | Give a cautious overall interpretation of results considering objectives, limitations, multiplicity of analyses, results from similar studies, and other relevant evidence | Page 9 |
| Limitations |  | Discuss limitations of the study, taking into account sources of potential bias or imprecision. Discuss both direction and magnitude of any potential bias | Page 10 |
|  |  |  |  |
|  |  |  |  |
| Other Information | 17. Conclusions | The main results correlated with the research objectives | Page 10 |
